# Review of the cardiovascular safety of dipeptidyl peptidase-4 inhibitors and the clinical relevance of the CAROLINA trial

**DOI:** 10.1186/s12872-019-1036-0

**Published:** 2019-03-15

**Authors:** Marile Santamarina, Curt J. Carlson

**Affiliations:** Gregory School of Pharmacy, Palm Beach Atlantic University, 901 South Flagler Drive, West Palm Beach, FL 33416 USA

**Keywords:** Type 2 diabetes mellitus, Cardiovascular diseases, DPP-4 inhibitors, Linagliptin, Sulfonylureas

## Abstract

**Background:**

Cardiovascular (CV) disease (CVD) is a well-recognized complication of type 2 diabetes mellitus (T2DM) and there is a clinical need for glucose-lowering therapies that do not further increase CV risk in this population. Although sulfonylureas (SUs) may be used as second-line therapy for patients requiring additional therapy after first-line metformin to improve glycemic control, their long-term effects on CV outcomes remain uncertain, and a wide range of alternative agents exist including dipeptidyl peptidase-4 (DPP-4) inhibitors.

**Methods:**

Literature searches in PubMed (2013–2018) were conducted with terms for DPP-4 inhibitors combined with CV terms, with preference given to cardiovascular outcomes trials (CVOTs). Reference lists from retrieved articles and diabetes guidelines were also considered.

**Results:**

This narrative review discusses current evidence for the CV safety of these agents, describes the long-term CV effects of DPP-4 inhibitors, including effects on CV events, mortality, the risk for heart failure hospitalization, and highlights the need for further research into the CV effects of SU therapy. Although SUs remain a treatment option for T2DM, the long-term effects of these agents on CV outcomes are unclear, and further long-term studies are required. For DPP-4 inhibitors, uncertainties have been raised about their long-term effect on hospitalization for heart failure in light of the results of SAVOR-TIMI 53, although the findings of other DPP-4 inhibitor CVOTs in T2DM and data analyses have suggested these agents do not increase the occurrence of adverse CV outcomes.

**Conclusions:**

Based on recent CVOTs and guideline updates, the choice of add-on to metformin therapy for patients with T2DM and established CV disease should be a sodium-glucose co-transporter-2 inhibitor or a glucagon-like peptide-1 agonist with proven CV benefit. Additional treatment options for those individuals who require therapy intensification, as well as in patients with T2DM and without established CVD include DPP-4 inhibitors and SUs. Since few head-to-head trials have compared the effects of different oral glucose-lowering agents on CV outcomes in T2DM, with most CVOTs using placebo as a comparator, the CAROLINA trial will provide important information on the comparative CV safety of a commonly prescribed SU and a DPP-4 inhibitor.

## Background

Cardiovascular (CV) disease (CVD) is a well-recognized complication of type 2 diabetes mellitus (T2DM), and is the leading cause of mortality and morbidity for patients with T2DM [1, 2]. In view of the risk of CVD in patients with T2DM and the need for long-term treatment, the choice of glucose-lowering therapy should prioritize agents that have recently been proven to reduce CV risk for patients with T2DM and established atherosclerotic CVD (ASCVD) or chronic kidney disease (CKD). For patients without established ASCVD or CKD, the choice of glucose-lowering therapy should take account of individual patient characteristics (e.g., hypoglycemia risk, effects on body weight), as well as cost and CV neutrality [1, 3].

The influence of many glucose-lowering agents on long-term CV risk was unclear until the recent availability of results from randomized placebo-controlled cardiovascular outcomes trials (CVOTs), which were recommended by the US Food and Drug Administration (FDA) over 10 years ago in response to CV safety concerns for glucose-lowering drugs [4]. Several CVOTs with published results have shown a neutral effect on ASCVD for dipeptidyl peptidase-4 (DPP-4) inhibitors compared with placebo when added to standard care for patients with T2DM and high CV risk or established CVD [5–8]. Moreover, the CAROLINA (CARdiovascular Outcome Study of LINAgliptin versus Glimepiride in Patients with Type 2 Diabetes; NCT01243424) trial has randomized 6041 patients with relatively early T2DM and increased CV risk or with established CV complications to receive once-daily therapy with either linagliptin 5 mg or glimepiride 1–4 mg, in addition to standard of care, and will assess the effect of each treatment on CV events [9, 10]. CAROLINA is the largest head-to-head comparison of an SU and a DPP-4 inhibitor, and will provide important additional evidence on the comparative long-term CV safety of these agents in T2DM.

CV outcomes have also been evaluated with other classes of glucose-lowering drugs, and important results have been reported for sodium-glucose co-transporter-2 (SGLT2) inhibitors and glucagon-like peptide-1 (GLP-1) receptor agonists. The Empagliflozin, Cardiovascular Outcomes, and Mortality in Type 2 Diabetes trial (EMPA-REG OUTCOME) [11], demonstrated lower rates of the composite 3-point major cardiovascular event (MACE) outcome of CV death, non-fatal myocardial infarction (MI), or non-fatal stroke (14% relative risk reduction); death from CV causes (38% relative risk reduction); death from any cause (32% relative risk reduction); and hospitalization for heart failure (35% relative risk reduction), with the SGLT2 inhibitor empagliflozin compared with placebo, when added to standard care [11]. The CANagliflozin cardioVascular Assessment Study (CANVAS) Program showed a reduction in the primary composite 3-point MACE outcome (hazard ratio [HR] 0.86; 95% confidence interval [CI] 0.75–0.97; *P* < 0.001 for non-inferiority; *P* = 0.02 for superiority); however, no statistically significant difference in CV mortality was observed (HR 0.87; 95% CI 0.72–1.06) [12]. The Dapagliflozin Effect on Cardiovascular Events–Thrombolysis in Myocardial Infarction 58 (DECLARE–TIMI 58) trial is the most recent and largest CVOT for the SGLT2 inhibitor class (*N* > 17,000). In this trial, dapagliflozin was associated with a lower rate of CV death or hospitalization for heart failure versus placebo (HR 0.83; 95% CI 0.73–0.95; *P* = 0.005) [13], primarily due to a 27% reduction in heart failure hospitalizations. Dapagliflozin treatment showed no effect on CV mortality and dapagliflozin was non-inferior, but not statistically superior compared with placebo, for 3-point MACE (HR 0.93; 95% CI 0.84–1.03; *P* = 0.17). Among the GLP-1 receptor agonist CVOTs, the Liraglutide Effect and Action in Diabetes: Evaluation of Cardiovascular Outcome Results (LEADER) trial showed a decreased risk of the 3-point MACE for liraglutide compared with placebo (HR 0.87; 95% CI 0.78–0.97; *P* < 0.001 for non-inferiority; *P* = 0.01 for superiority) [14]. Also, a non-inferiority study with semaglutide showed that among patients with T2DM and high CV risk, the occurrence of 3-point MACE was lower among patients receiving semaglutide versus placebo, when added to standard care [15].

In response to the findings of recent CVOTs, treatment recommendations for T2DM have been updated. Current consensus statements from the American Diabetes Association (ADA) [3], the ADA and the European Association for the Study of Diabetes (EASD) [16], and the American Association of Clinical Endocrinologists (AACE) and American College of Endocrinology (ACE) [17] recommend metformin as first-line therapy in the treatment of individuals with T2DM. For patients with T2DM and ASCVD, the latest guidelines from the ADA and ADA/EASD recommend addition of an SGLT2 inhibitor or a GLP-1 receptor agonist with proven CV benefit [1, 16]. For patients with ASCVD and a high risk of heart failure or coexisting heart failure, SGLT2 inhibitors might be appropriate [1]. In individuals with T2DM without established ASCVD or CKD, the choice of second-line therapy is based on minimizing risks, especially hypoglycemia and weight gain, cost, and patient preference. If subsequent third-line glucose-lowering therapies are required, similar principles apply. Several treatment options are available, including DPP-4 inhibitors [3, 17], and sulfonylureas (SUs); the latter remain an option when cost is a major consideration [3]. SUs were the first available oral glucose-lowering drugs, and have been in clinical use since the late 1950s [18, 19]. The low cost and familiarity of SUs in clinical practice are possible factors underlying their continued use in T2DM [18, 20].

The aim of this review is to provide a summary of current evidence for the long-term CV safety of DPP-4 inhibitors and the role of further research into the long-term CV effects of these agents. We also consider the available CV safety data for SUs, and the importance of the CAROLINA trial as the largest head-to-head study to evaluate the relative safety of a DPP-4 inhibitor and an SU.

## Methods

The Medline database was searched via PubMed to retrieve relevant articles on DPP-4 inhibitors between 2013 and 2018 (limits: English language, humans). The search results were combined with CV-related terms, and cardiovascular outcome trial-related terms (e.g., TECOS, SAVOR). Other relevant literature was obtained based on personal knowledge and experience, including evidence from cardiovascular outcomes trials, meta-analyses, and systematic literature reviews. A narrative overview of the literature was then synthesized based on manual assessment of the retrieved literature.

## Results

### Published evidence from clinical trials with SUs or DPP-4 inhibitors

There is uncertainty surrounding the overall CV safety of SUs in the treatment of T2DM. Since the publication of a study by the University Group Diabetes Program in 1970, concerns have been raised about the long-term CV safety of SUs after an increased occurrence of CV mortality was observed among SU-treated patients versus patients managed with diet alone or diet plus insulin [21]. The tolbutamide arm of the study was stopped early due to the increased number of CV deaths versus placebo that were observed at an interim analysis of data during the study. In response to this finding, the FDA included a warning in the prescribing information of all US-marketed SUs about the 2.5-fold increased risk of CV mortality associated with SU therapy in this study [22, 23].

More recent evidence has also suggested a link between SU therapy and an increased risk of CV events and mortality [24], although controversy remains [10, 25, 26]. In two large trials of long-term treatment of T2DM, in which SUs were widely used in the intensive glucose-lowering arms, no increased CV risk was reported [27, 28]. The UK Prospective Diabetes Study (UKPDS) reported that after 10 years of intensive glucose-lowering therapy, no adverse effects on CV outcomes were observed with the three agents (two first-generation SUs, chlorpropamide and glibenclamide, or insulin) [28]. Similarly, the Action in Diabetes and Vascular Disease: Preterax and Diamicron MR Controlled Evaluation (ADVANCE) study showed no significant differences in the rate of death from CV causes, over a median follow-up of 5 years, among patients who received intensive glucose control (involving modified-release gliclazide with target glycated hemoglobin [HbA1c] of ≤6.5%) versus standard glycemic control (HbA1c target based on local guidelines) [27]. In addition, the long-term, randomized Thiazolidinediones Or Sulphonylureas and Cardiovascular Accidents. Intervention Trial (TOSCA.IT) showed that the incidence of CV events was similar with SUs (mostly glimepiride and gliclazide) and pioglitazone, when added to existing metformin therapy, over a median follow-up period of 57.3 months [29]. However, in the Action to Control Cardiovascular Risk in Diabetes (ACCORD) study, intensive glucose control was associated with an increase in all-cause mortality and CV mortality compared with standard therapy (SUs were used in more than half of patients in both groups) over a mean follow-up period of 3.5 years [24]. In addition, the findings of several retrospective cohort studies using the ACCORD data have suggested an increased risk of adverse CV outcomes associated with SU therapy compared with metformin [30–32]. A retrospective cohort study of patients with T2DM newly treated with SUs versus metformin showed an increased risk of all-cause and CV mortality among patients newly treated with SU monotherapy compared with metformin, with respective adjusted relative risks of 1.43 (95% CI 1.15–1.77) and 1.70 (95% CI 1.18–2.45) [32]. Another retrospective cohort study showed significantly higher risks of all-cause mortality (HR 1.46; 95% CI 1.21–1.76) and major adverse CV events (HR 1.58; 95% CI 1.19–2.10) for SU monotherapy compared with metformin plus SU combination therapy [31]. A further study indicated a higher risk of mortality (of which around one-fourth of deaths were due to an acute ischemic event) with first-generation SUs (adjusted HR 2.1; 95% CI 1.0–4.7 for patients receiving doses higher than the median) and second-generation SUs (glyburide, adjusted HR 1.3; 95% CI 1.2–1.4 for higher doses), with a relationship between greater daily dose and increased risk of mortality [30]. Evidence from other observational studies has indicated either an increased risk of CV events and/or mortality associated with SU therapy [33, 34] or no increased risk [35–37]. However, this evidence is largely based on retrospective analysis and it remains unclear whether SU use directly contributes to an increased risk of CV events [26], and whether this is more likely to occur with first- versus second-generation SUs [10, 38]. A recent review of CV outcomes reported in meta-analyses of randomized controlled trials and observational studies involving SUs suggested that overall the risk of CV events and mortality is higher in patients with T2DM treated with SUs versus other types of glucose-lowering agents (including DPP-4 inhibitors) or placebo, albeit given that such analyses have limitations [39] (Table 1).

**Table 1:** Summary of the effects of DPP-4 inhibitors and SU agents on CV outcomes

Clinical trial	Intervention	Primary outcome	CV risk	HR, OR or RR (95% CI)
*DPP-4 inhibitors*				
EXAMINE [8] *N* = 5380	Alogliptin versus placebo	3-point MACE	↔	HR 0.96 (≤ 1.16)^a^
SAVOR-TIMI 53 [7] *N* = 16,492	Saxagliptin versus placebo	3-point MACE	↔	HR 1.00 (0.89–1.12)
TECOS [5] *N* = 14,671	Sitagliptin versus placebo	4-point MACE	↔	HR 0.98 (0.88–1.09)
CARMELINA [6] *N* = 6979	Linagliptin versus placebo	3-point MACE	↔	HR 1.02 (0.89–1.17)^b^
CAROLINA *N* = 6041	Linagliptin versus glimepiride	3-point MACE	c [lc]	c [lc]
*SUs* ^d^				
Rados et al., 2016 [92] MA, 47 RCTs ≥52 wk	SUs (2nd/3rd generation only) versus other comparators	All-cause mortality	↔	P-OR 1.12 (0.96–1.30)
		CV mortality	↔	P-OR 1.12 (0.87–1.42)
Monami et al., 2013 [93] MA, 115 RCTs (62 reported MACE), ≥24 wk	All SUs versus other comparators	MACE^e^	↔	MH-OR 1.08 (0.86–1.36)^f^
		Mortality	↑	MH-OR 1.22 (1.01–1.49)^g^
Phung et al., 2013 [94] MA, 12 RCTs, 21 Observational, 6 mo to 10 yr	All SUs versus other comparators	4-point MACE	↑	RR 1.10 (1.04–1.16)
		RCTs only	↔	RR 0.98 (0.73–1.32)
		Observational only	↑	RR 1.11 (1.05–1.18)
		CV mortality	↑	RR 1.27 (1.18–1.34)
		RCTs only	↔	RR 1.22 (0.63–2.39)
		Observational only	↑	RR 1.26 (1.18–1.34)

^a^Upper boundary of one-sided repeated CI

^b^*P* < 0.001 for non-inferiority

^c^Trial completed; publication of final data awaited

^d^There are no dedicated CVOTs for SUs; data are taken from meta-analyses of randomized clinical trials and observational studies involving SUs

^e^Defined by Monami et al. as CV death, non-fatal myocardial infarction, stroke, acute coronary syndromes, and/or heart failure reported as serious adverse events

^f^*P* = 0.52

^g^*P* = 0.047

↑ = increase in CV risk; ↔ = neutral effect on CV risk; *CV* cardiovascular, *CVOT* cardiovascular outcomes trial, *HR* Hazard ratio, *MA* meta-analysis, *MACE* major adverse cardiovascular event (3-point: CV death, non-fatal MI, or non-fatal stroke; 4-point: 3-point MACE plus hospitalization for unstable angina), *MH-OR* Mantel-Haenzel odds ratio, *P-OR* Peto odds ratio, *RCT* randomized clinical trial, *RR* relative risk, *SU* sulfonylurea

SUs are also generally regarded as having the highest risk of hypoglycemia of any non-insulin therapy [17, 40]. The elevated incidence of hypoglycemia with SU therapy is related to its mode of action, which involves stimulation of insulin release from pancreatic beta cells that occurs independently of plasma glucose levels [41]. Hypoglycemia is recognized as an important clinical complication with these agents [3, 17], and the overall cost of SU therapy could be underestimated if the health care financial burden of treatment of hypoglycemic events are not taken into account [42, 43]. Patients receiving SUs are more likely than those treated with newer agents, such as DPP-4 inhibitors, to experience severe hypoglycemic episodes requiring hospital treatment, adding substantial health care costs to the care of patients with T2DM [43]. The occurrence of hypoglycemic events is a particular risk for elderly patients [44], for whom the additional risks of falls and fractures are also a concern, adding to the clinical and economic burden of hypoglycemia.

Another important consequence of severe hypoglycemia is an approximately 2-fold increased risk of CV events and mortality [45–47] that can also lead to an elevated incidence of hospital admission and related health care costs [48, 49]. The association of severe hypoglycemia and CV events is not entirely explained by the presence of comorbid illness [45], and several possible mechanisms have been suggested to underlie this observation. Hypoglycemia has been described as a pro-arrhythmic, pro-inflammatory and pro-thrombotic state that could lead to vascular changes associated with CVD [50, 51]. Furthermore, prolongation of the QT interval has been demonstrated during episodes of hypoglycemia, increasing the risk of arrhythmia and sudden death at low blood glucose levels [52, 53]. A link between hypoglycemia and the occurrence of myocardial ischemia has also been demonstrated, particularly in patients who experience substantial fluctuations in blood glucose levels [54]. It has also been suggested that hypoglycemic episodes can lead to impaired autonomic function, which contributes to increased mortality in patients with T2DM and CVD [55]. The avoidance of hypoglycemia, therefore, may be an important component of reducing the risk of adverse CV events and mortality in patients with T2DM [45]. It remains unclear whether a high frequency of severe hypoglycemic events is a marker of poor health in patients with existing high CV risk or whether the hypoglycemia itself is a causal factor for adverse CV events [46, 50, 56–58]. The findings of recent clinical trials have added to the controversy surrounding hypoglycemia and CV events. In TOSCA.IT, second-line treatment with SUs (glimepiride [48%], gliclazide [50%] or glibenclamide [2%]) was compared with pioglitazone over a median of 4.8 years (57.3 months). A similar incidence of CV events was observed in both groups, despite more frequent occurrence of hypoglycemic events in the SU group [29]. Further uncertainty about the relationship between severe hypoglycemia and CV events was raised in a post hoc analysis of data from the Trial Evaluating Cardiovascular Outcomes with Sitagliptin (TECOS), in which participants with T2DM and CVD were randomized to receive sitagliptin or placebo in addition to standard-of-care therapy [5] (almost half of patients in both groups were receiving background SU therapy) [59]. Although DPP-4 inhibitor therapy was not associated with an increase in the occurrence of hypoglycemic events, the analysis demonstrated a greater risk of major CV events following episodes of severe hypoglycemia, and also almost twice the risk of severe hypoglycemic episodes among patients who experienced a previous major CV event [59]. The authors suggest these findings indicate that severe hypoglycemia could be a marker of patient frailty rather than a cause of increased CV events or CV mortality.

Independent of the effects of hypoglycemia, a suggested mechanism for the potential adverse CV effects of SU therapy relates to their potential to block ion channels within the myocardium including the K_ATP_ channel [60]. Blockade of K_ATP_ channels is thought to underlie the mode of action of SUs in pancreatic beta cells, however it is unclear whether blockade of these channels in the myocardium leads to detrimental effects in patients with T2DM at risk for CVD [60]. SU-induced impairment of the vasodilatory response to myocardial ischemia has been postulated [61]. Furthermore, it is possible that findings with older, first-generation SUs do not apply to newer agents [60]. There is evidence to suggest that unlike other SUs, glimepiride does not impair the myocardial protection induced by ischemic preconditioning, an effect in which brief periods of myocardial ischemia, non-lethal to cardiac myocytes, decreases injury to cardiac muscle during subsequent longer term ischemia [62]. This apparent benefit over the other SUs indicates that comparison of glimepiride with other second-line treatments for T2DM would be of great interest and relevant to current clinical practice [63, 64]. In the absence of convincing evidence of a causal association between SU use and an increased risk of CV events or mortality, further research is clearly required, and there remains a clinical need for approaches to glycemic control that do not further increase CV risk [65].

### CV safety of DPP-4 inhibitors

CVOTs involving DPP-4 inhibitors were conducted in a manner to allow the patients’ physicians to adjust their diabetes therapies as needed to maintain glycemic targets generally in accordance with national or regional guidelines, although the exact definition of standard care varied. The Saxagliptin Assessment of Vascular Outcomes Recorded in Patients with Diabetes Mellitus – Thrombolysis in Myocardial Infarction 53 (SAVOR-TIMI 53) [7], Examination of Cardiovascular Outcomes With Alogliptin Versus Standard of Care (EXAMINE) [8] and TECOS [5] trials demonstrated that the addition of saxagliptin, alogliptin and sitagliptin, respectively, had a neutral effect (i.e., did not increase or decrease the event rate versus placebo) on the primary composite endpoint (defined as CV death, non-fatal stroke or non-fatal MI) in SAVOR-TIMI 53 and EXAMINE, and as CV death, stroke, MI or hospitalization for unstable angina in TECOS) in patients with high baseline risk for CV events (Table 1). SAVOR-TIMI 53 [7] demonstrated that the addition of saxagliptin to existing therapy had a neutral effect on individual components of the primary composite endpoint (namely the risk of CV death, non-fatal MI or non-fatal ischemic stroke) versus placebo; however, saxagliptin treatment was associated with a significantly increased risk of heart failure-related hospital admissions. After concerns raised by the findings of SAVOR-TIMI 53 [7], several studies have investigated the effect of DPP-4 inhibitors on the risk of heart failure in patients with T2DM. The EXAMINE trial [8] showed that the addition of alogliptin to standard care in patients with T2DM and a history of recent acute coronary syndrome was not associated with an increase in major CV events versus placebo, although a post hoc analysis showed a non-significant 19% increased risk of hospitalization for heart failure among alogliptin- versus placebo-treated patients [66]. In TECOS, no increases in major adverse CV events or hospitalization for heart failure were observed for sitagliptin versus placebo [5]. Subsequently, a retrospective observational study that analyzed data from a US insurance claims database (Truven Health MarketScan Commercial and Medicare Supplemental databases) found no association between hospitalization for heart failure and treatment with a DPP-4 inhibitor relative to SU therapy, or treatment with saxagliptin relative to sitagliptin [67]. Although the causal mechanism for the increased risk of heart failure observed with DPP-4 inhibitor therapy in SAVOR-TIMI 53 and EXAMINE is currently unknown, sympathetic nervous system activation resulting in cardiomyocyte injury and death has been proposed as a possible explanation, as the actions of stromal derived factor-1, neuropeptide Y, and substance P to stimulate β-adrenergic receptors and cause cardiomyocyte apoptosis may be potentiated by DPP-4 inhibition [68].

More recently, the Cardiovascular and Renal Microvascular Outcome Study with Linagliptin in Patients with Type 2 Diabetes Mellitus (CARMELINA) trial has demonstrated the CV and renal safety of linagliptin versus placebo when added to standard care in patients with T2DM who were at high risk of vascular complications [6]. Participants were generally at high vascular risk at baseline, with long-standing T2DM (mean duration, 14.7 years) and established CKD, with or without CVD [69]. Compared with placebo, the addition of linagliptin to standard care over a median of 2.2 years did not increase the risk of the composite CV outcome of 3-point MACE (CV death, non-fatal MI, non-fatal stroke) (HR 1.02; 95% CI 0.89–1.17). There was also no increase in risk of the kidney composite outcome of renal death, end-stage kidney disease, or sustained ≥40% decrease in eGFR from baseline (HR 1.04; 95% CI 0.89–1.22). Importantly, linagliptin did not increase the risk of hospitalization for heart failure (HR 0.90; 95% CI 0.74–1.08). This study provides new information on the safety of linagliptin administration in a population at high cardio-renal risk who generally have limited treatment options, and have been under-represented in previous CVOTs in patients with T2DM [6, 69].

The data from the DPP-4 inhibitor CVOTs contrasted with the results of pooled analyses and meta-analyses of smaller trials that had suggested a reduction in the risk of major CV events was associated with DPP-4 inhibitor treatment [70]. For example, a meta-analysis of 36 placebo-controlled randomized clinical trials of DPP-4 inhibitors reported no significant differences in all-cause mortality (relative risk reduction 1.03; 95% CI 0.95–1.12) and CV mortality (relative risk reduction 1.02; 95% CI 0.92–1.12) with the use of these agents [71]. Similar results were reported in other meta-analyses of DPP-4 inhibitor studies [72, 73]. Whereas an earlier meta-analysis of 70 randomized clinical trials involving DPP-4 inhibitors stated that treatment with DPP-4 inhibitors reduced the risk of CV events, as the Mantel-Haenzel odds ratio for MACE (defined here as CV death, non-fatal MI, stroke, acute coronary syndromes, and/or heart failure reported as serious adverse events) was 0.71 (95% CI 0.59–0.86) [74]. However, these results are not surprising given that these trials were designed to assess metabolic rather than CV endpoints, most had durations below 2 years, management of concurrent medications varied, and they utilized a heterogeneous study population of T2DM patients with a generally low risk of CV events [39]. The differences in CV outcomes between the CVOTs and meta-analyses of “non-CVOTs” for DPP-4 inhibitors may be largely explained by these differences in study and patient characteristics [70].

### DPP-4 inhibitor selection: Focus on linagliptin

Linagliptin, a DPP-4 inhibitor with an established role in the management of T2DM, has a unique pharmacology within its drug class. One distinctive characteristic of linagliptin is that its route of elimination is via the bile and gut, unlike the other currently available DPP-4 inhibitors that undergo mainly renal excretion [75, 76]. Thus, no dosage adjustment is required for linagliptin in patients with CKD, whereas sitaglptin, saxagliptin, and alogliptin may be used in renal impairment with dosage adjustment [3]. In contrast to other CVOTs in the DPP-4 inhibitor class, the CARMELINA trial included a substantial proportion of patients with T2DM; 74% had prevalent kidney disease [6]. As discussed above, linagliptin was non-inferior in the primary 3-point MACE outcome when compared with placebo in patients with T2DM and established CVD or CKD on top of standard-of-care treatment [6]. The CV safety of linagliptin was previously evaluated in three pooled analyses of data from the clinical trial program. A pooled analysis of 19 randomized, controlled trials showed that the incidence of prospectively adjudicated CV events was similar for linagliptin versus pooled active comparators or placebo [77]. Randomized clinical trials have demonstrated that linagliptin, both alone and when used in combination with other glucose-lowering agents, is effective and well tolerated, with a low incidence of hypoglycemia [78–81]. Also, a post hoc pooled analysis of four randomized clinical trials of linagliptin added on to insulin therapy for T2DM demonstrated a neutral effect of linagliptin on the occurrence of major CV events [82]. A further pooled analysis of safety data from 19 trials evaluated a high-risk patient population with T2DM and pre-existing coronary artery disease and showed that the addition of linagliptin to existing treatment was not associated with an increase in cardiac adverse events [83]. A 2-year study of linagliptin versus glimepiride added to metformin therapy in patients with inadequately controlled T2DM showed significantly fewer CV events among linagliptin-treated patients compared with those receiving glimepiride (12 vs 26 patients; relative risk 0.46; 95% CI 0.23–0.91) [84]. However, this study was neither powered nor designed to evaluate the effect of linagliptin or glimepiride on CV events, and the low number of CV events reported in this study preclude drawing any definitive conclusions.

Further information on the comparative CV safety of linagliptin versus the SU, glimepiride, will be provided with the results of the CAROLINA trial. Baseline demographic data from CAROLINA show that patients had a diagnosis of T2DM for a median of 6.2 years (with 40.6% ≤ 5 years) at entry into the study. This is a shorter duration than in the other DPP-4 inhibitor CVOTs (T2DM duration: median 10.3 years in SAVOR-TIMI 53; median 7.1 years in EXAMINE; mean 11.6 years in TECOS and mean 14.7 years in CARMELINA). It remains to be seen whether intervention at an earlier stage in the disease will have an important impact on CV and other outcomes in the long term.

## Discussion

### Clinical implications of the CAROLINA trial

The management of T2DM presents unique challenges largely due to the progressive nature of the condition, the frequent presence or development of comorbidities, and the need for the use of additional agents over time that incorporate the individual needs of the patient. At present, treatment choices are based on consideration of a range of factors including efficacy, low risk of hypoglycemia, effect on body weight, and overall safety data. More recently, the importance of the reduction of CV risk has been emphasized [1, 17] in light of the findings of CVOTs that have shown CV risk reduction with SGLT2 inhibitors [11, 12, 85] and GLP-1 receptor agonists [14, 15], and the latest ADA recommendations state that second-line therapy for patients with established CVD should include an agent proven to reduce major adverse CV events and CV mortality (currently SGLT2 inhibitors or GLP-1 agonists) [1]. These agents also have been shown to reduce the risk of secondary outcomes such as heart failure and progression of renal disease, and therefore treatment guidelines now recommend that the presence of ASCVD, heart failure and CKD be taken into account when making treatment choices [1]. To further assist with the selection of appropriate treatments for individuals with T2DM, there is a need for head-to-head clinical trials to evaluate the CV safety and efficacy of glucose-lowering agents in long-term clinical use.

Although SUs remain a treatment option for T2DM [3, 17], the long-term effects of these agents on CV outcomes are uncertain. Furthermore, there is some evidence to suggest that the therapeutic durability of SUs is unreliable, with a decline in efficacy of these agents (in terms of fasting plasma glucose and HbA1c levels) demonstrated over long-term follow-up [29, 86, 87]. SUs consistently demonstrate a progressive decline in glycemic control after the initial 18 months of treatment [88, 89], with data from the UKPDS indicating that glycemic control with SU monotherapy becomes inadequate (fasting plasma glucose > 15.0 mmol/L or development of hyperglycemic symptoms) in 44% of patients after 5 years of treatment [90]. A well-recognized problem with SU therapy is the risk of hypoglycemic events [3, 17]. As stated previously, the prognostic clinical implications of severe hypoglycemia associated with SU therapy is currently unclear, although it appears that its occurrence might be an indicator of deteriorating health and could indicate the need for increased monitoring of affected patients. Further research will help shed light on the question of whether a causal link exists between severe hypoglycemia and increased CV events and mortality. This has important clinical implications for the selection of T2DM treatments; if indeed the occurrence of hypoglycemia is a marker of patient vulnerability to CV events, the selection of agents on the basis of hypoglycemia risk alone might not result in an accompanying reduction in the risk of CV events or mortality.

The DPP-4 inhibitors are newer agents among the recommended options for second-line therapy in T2DM [3, 17]. Although uncertainties were raised about the long-term effect of DPP-4 inhibitors on hospitalization for heart failure following publication of the results of SAVOR-TIMI 53 [7], the findings of other DPP-4 inhibitor CVOTs in T2DM [5, 8], including the recently published CARMELINA trial [6], in addition to retrospective evaluation [67] and pooled analyses [77, 82, 83] of these agents, have provided some reassurance. To date, no head-to-head trials have compared the effects of different oral glucose-lowering agents on CV outcomes in patients with T2DM [1, 91]. The CAROLINA trial will provide an important opportunity to evaluate the comparative CV safety of a commonly prescribed SU and a DPP-4 inhibitor and, thus, CAROLINA will be the first CVOT to provide a head-to-head comparison of a DPP-4 inhibitor with another class of oral glucose-lowering agent.

## Conclusions

Recent evidence and guideline updates indicate that the choice of add-on to metformin therapy should be based on consideration of agents with proven CV risk reduction (currently SGLT2 inhibitors or GLP-1 agonists). When additional glucose-lowering is required, DPP-4 inhibitors and SUs may be considered, and the results of the CAROLINA trial will provide evidence on the comparative long-term CV safety of these agents.

## Abbreviations

AACE: American Association of Clinical Endocrinologists
ACCORD: Action to Control Cardiovascular Risk in Diabetes
ACE: American College of Endocrinology
ADA: American Diabetes Association
ADVANCE: Action in Diabetes and Vascular Disease: Preterax and Diamicron MR Controlled Evaluation
ASCVD: Atherosclerotic cardiovascular disease
CARMELINA: Cardiovascular and Renal Microvascular Outcome Study with Linagliptin in Patients with Type 2 Diabetes Mellitus
CAROLINA: CARdiovascular Outcome Study of LINAgliptin versus Glimepiride in Patients with Type 2 Diabetes
CI: Confidence interval
CKD: Chronic kidney disease
CV: Cardiovascular
CVD: Cardiovascular disease
CVOT: Cardiovascular outcomes trial
DECLARE–TIMI 58: Dapagliflozin Effect on Cardiovascular Events–Thrombolysis in Myocardial Infarction 58
DPP-4: Dipeptidyl peptidase-4
EASD: European Association for the Study of Diabetes
EMPA-REG OUTCOME: Empagliflozin, Cardiovascular Outcomes, and Mortality in Type 2 Diabetes
EXAMINE: Examination of Cardiovascular Outcomes With Alogliptin Versus Standard of Care
FDA: US Food and Drug Administration
GLP-1: Glucagon-like peptide-1
HbA1c: Glycated hemoglobin
HR: Hazard ratio
LEADER: Liraglutide Effect and Action in Diabetes: Evaluation of Cardiovascular Outcome Results
MACE: Major adverse cardiovascular event
MI: Myocardial infarction
SAVOR-TIMI 53: Saxagliptin Assessment of Vascular Outcomes Recorded in Patients with Diabetes Mellitus – Thrombolysis in Myocardial Infarction 53
SGLT2: Sodium-glucose co-transporter-2
SU: Sulfonylurea
T2DM: Type 2 diabetes mellitus
TECOS: Trial Evaluating Cardiovascular Outcomes with Sitagliptin
TOSCA.IT: Thiazolidinediones Or Sulphonylureas and Cardiovascular Accidents.Intervention Trial)
UGDP: University Group Diabetes Program
UKPDS: UK Prospective Diabetes Study
